# Microbial Population Changes and Their Relationship with Human Health and Disease

**DOI:** 10.3390/microorganisms7030068

**Published:** 2019-03-03

**Authors:** Ana Isabel Álvarez-Mercado, Miguel Navarro-Oliveros, Cándido Robles-Sánchez, Julio Plaza-Díaz, María José Sáez-Lara, Sergio Muñoz-Quezada, Luis Fontana, Francisco Abadía-Molina

**Affiliations:** 1 Department of Biochemistry and Molecular Biology II, School of Pharmacy, University of Granada, 18071 Granada, Spain; croblesan@hotmail.com (C.R.-S.); fontana@ugr.es (L.F.); 2 Institute of Nutrition and Food Technology “José Mataix,” Center of Biomedical Research, University of Granada, Avda. del Conocimiento s/n. 18016 Armilla, Granada, Spain; miguelno@ugr.es; 3 Instituto de Investigación Biosanitaria IBS.GRANADA, Complejo Hospitalario Universitario de Granada, 18014 Granada, Spain; 4 Department of Biochemistry and Molecular Biology I, School of Sciences, University of Granada, 18071 Granada, Spain; mjsaez@ugr.es; 5 Departamento de Farmacia, Facultad de Química y de Farmacia, Pontificia Universidad Católica de Chile, Santiago 6094411, Chile; chechomu@hotmail.com; 6 National Agency for Medicines (ANAMED), Public Health Institute, Santiago 7780050, Chile; 7 Department of Cell Biology, School of Sciences, University of Granada, 18071 Granada, Spain

**Keywords:** gut microbiota, microbial population changes, randomized clinical trial, health status, obesity, non-communicable diseases, non-alcoholic fatty liver disease, inflammatory bowel disease

## Abstract

Specific microbial profiles and changes in intestinal microbiota have been widely demonstrated to be associated with the pathogenesis of a number of extra-intestinal (obesity and metabolic syndrome) and intestinal (inflammatory bowel disease) diseases as well as other metabolic disorders, such as non-alcoholic fatty liver disease and type 2 diabetes. Thus, maintaining a healthy gut ecosystem could aid in avoiding the early onset and development of these diseases. Furthermore, it is mandatory to evaluate the alterations in the microbiota associated with pathophysiological conditions and how to counteract them to restore intestinal homeostasis. This review highlights and critically discusses recent literature focused on identifying changes in and developing gut microbiota-targeted interventions (probiotics, prebiotics, diet, and fecal microbiota transplantation, among others) for the above-mentioned pathologies. We also discuss future directions and promising approaches to counteract unhealthy alterations in the gut microbiota. Altogether, we conclude that research in this field is currently in its infancy, which may be due to the large number of factors that can elicit such alterations, the variety of related pathologies, and the heterogeneity of the population involved. Further research on the effects of probiotics, prebiotics, or fecal transplantations on the composition of the human gut microbiome is necessary.

## 1. Introduction

Health is defined as “the state of the organism when it functions optimally without evidence of disease,” and the words “microbes” or “microorganism” are surprisingly missing in this definition. Currently, sequencing technologies (e.g., next-generation sequencing technologies) have stimulated new research that relates the microbial communities that reside in our gut with a number of physiological conditions. In 2014, the term microbiota was defined as the “full collection of microbes (bacteria, fungi, and viruses, among others) that naturally exist within a particular biological niche,” an estimated 500–1000 species [1,2,3] that may have a tremendous impact on human health.

The gut microbiota is regulated by an enormous number of factors, such as microbiological factors, host characteristics, diet patterns, and environmental variables [4]. Some protective, structural, and metabolic functions have been reported for gut microbiota [5], and these functions are associated with the regulation of homeostasis and host health. Host defenses against pathogens are in part mediated through the activity of the gut microbiota, requiring an intimate interpretation of the current microenvironment to discriminate between commensal and transient bacteria [6,7].

The intestinal epithelium is constantly exposed to high levels of food and bacterial antigens. Under normal physiological conditions, the intestinal epithelial monolayer facilitates a controlled and selective flux of components between the lumen and the underlying mucosa [8]. The intestine and the gut-associated lymphoid tissue are essential components of the immune defense system, protecting the host from foreign antigens and pathogens while tolerating commensal bacteria and dietary antigens. Antigen-presenting cell populations in the gut dictate in part the homeostasis between tolerance and immunity in the intestine, and the dysregulation of this balance can contribute to the pathogenesis of numerous inflammatory conditions [9]. All of the aforementioned cells are involved in the host response, and their function principally involves the maintenance of homeostasis.

Endogenous and exogenous factors influence the gut microbiota [10,11] including the mode of delivery of a neonate [12], host genetic features [13], host immune response [14], diet [15] (including dietary supplements, breast-feeding, and formula-feeding), xenobiotics (including antibiotics) and other drugs [16,17], infections [18], diurnal rhythm [19], and environmental microbial exposures [20,21]. Despite evidence linking dysbiosis of the gut microbiota with disease manifestations at sites distant from the gut, most studies have not explored mechanisms outside the affected site, nor have they considered the effect of the microbiota and its varied products on the multitude of molecular pathways potentially involved [21]. Overall, our current understanding of the precise relationships between the human gut microbiome and disease remains limited. Case–control studies often report disease-associated microbial dysbiosis, defined as the alteration in terms of the diversity, quantity, and stability of resident commensal communities relative to the community found in healthy individuals [3,22]. 

Probiotics are defined as “live microorganisms that confer a health benefit to the host when administered in adequate amounts, although dead bacteria and bacterial molecular components may also exhibit probiotic properties” [23], whereas a prebiotic is a non-viable food component that confers a health benefit to the host and is associated with the modulation of the intestinal microbiota [3]. The combination of prebiotics and probiotics is referred to as synbiotics [3,24,25].

A healthy host–microorganism balance contributes to optimally perform metabolic and immune functions as well as prevent disease development. Indeed, alterations in the delicate host–microbe relationship is nowadays recognized as associated with a wide variety of diseases such as cancer, neurological, ophthalmological, premature newborn, extra-intestinal and intestinal diseases, and metabolic disorders, such as non-alcoholic fatty liver disease and type 2 diabetes (T2D) [26]. However, the specific changes in the intestinal microbiome that are associated with these pathologies remain unelucidated.

To shed some light on this matter, a comprehensive search of the relevant literature reported during the last five years was performed in multiple electronic databases, including MEDLINE (PubMed), EMBASE, and the Cochrane Library. MEDLINE was searched through PubMed for scientific articles in English using the terms “microbiota” combined with “obesity,” “inflammatory bowel disease,” “non-alcoholic fatty liver disease,” “insulin resistance syndrome,” and “diabetes mellitus type 2”. The gut microbiota changes associated with the following conditions are reviewed: (1) obesity—antibiotics and obesity, gut microbiota and children’s obesity, bariatric surgery and the gut microbiota, potential gut microbiota biomarkers in obesity, and clinical trials; (2) inflammatory bowel disease—ulcerative colitis, and Crohn’s disease; (3) non-alcoholic fatty liver disease; (4) insulin resistance syndrome; and (5) diabetes mellitus type 2. 

## 2. Gut Microbiota Changes in Particular Pathologies

### 2.1. Obesity

The prevalence of obesity is increasing worldwide [27,28] and currently represents a major health problem in both adults and children. The etiology of obesity has been associated with diverse factors, such as dietary, environmental, educational, and genetic factors. However, these factors do not fully explain the global incremental rise in obesity, and microbiota traits have recently been shown to play a causative role in obesity [29,30], demonstrating the potential of the microbiota as a therapeutic target in obesity. Here we present evidence-based studies linking host gut bacteria to obesity.

#### 2.1.1. Antibiotics and Obesity

Antibiotic exposure has an overwhelming impact on the gut microbiota [31]. Epidemiological studies have shown that the administration of antibiotics during childhood is associated with a major risk of obesity [32,33]. Well-known studies have demonstrated the correlation between obesity and the intestinal microbiota balance [34,35]. These investigations showed an obesity-associated decrease in the phylum Bacteroidetes and an increase in the phylum Firmicutes, alterations in the gut microbiota that have been confirmed in animal models that exhibit fat accumulation due to an alteration of host metabolism [36,37]. Early life exposure to antibiotics has also been suggested to lead to obesity [38]. Interestingly, a decrease in the number of antiobesogenic bacteria (bifidobacteria and *Bacteroides*) after repeated exposure to antibiotics in neonates has been described [39]. Moreover, several studies have reported a correlation between childhood body mass index (BMI) and the risk of developing obesity later in life with antibiotics consumption [32,33,40,41,42,43]. Clearly, as revealed from extensive cohort, multicenter studies, gut microbiome alterations induced by antibiotics may promote obesity in adulthood, emphasizing the need for additional research on the relationships among antibiotic exposure, the intestinal microbiota balance, and the susceptibility to develop obesity.

#### 2.1.2. Gut Microbiota and Childhood Obesity

The aforementioned alterations in gut bacteria have been very recently confirmed in children. An elevated abundance of Firmicutes and a decreased abundance of Bacteroidetes have been shown to correlate with overweight and obesity [44]. Interestingly, Bacteroidetes have been shown to be a better predictor of BMI than Firmicutes, possibly due to a higher variability of Firmicutes. This phenomenon may explain the results of a microbiome profiling study of eight obese children in whom the phylum Bacteroidetes exhibited the aforementioned tendency while Firmicutes showed little variation compared to the controls [45]. The levels of short chain fatty acids (SCFAs), the end products of fermentation of dietary fibers by the anaerobic intestinal microbiota, have been shown to be higher in obese children [44,46,47], suggesting that gut dysbiosis and augmented intestinal fermentation must be considered to be among the factors involved in the etiology of infant obesity. Interestingly, the use of prebiotics has been shown in a recent study to alter the intestinal microbiota and reduce body fat in obese or overweight children [48]. Personalized nutrition [49,50], the use of prebiotics, probiotics, postbiotics, and synbiotics [51], fecal microbiota transplantation (FMT) [52,53], dietary education [54], and physical activity [55] constitute the primary approaches aimed at using the gut microbiota as a therapeutic target for obesity in children. In a prospective study of 70 children within a four-year window, pre-obese dysbiotic gut and dietary habits were correlated with excessive weight gaining [56].

#### 2.1.3. Bariatric Surgery and the Gut Microbiota

The gut microbiota has been shown to mediate the metabolic benefits and weight loss observed after bariatric surgery. Bariatric surgery alters the expression of several genes involved in metabolic pathways, which induces changes in the gut microbiota composition [57,58,59,60]. Given the relationship between the gut microbiota and the metabolic health of an individual, there is a growing interest in understanding the modifications that occur in intestinal microbiota after bariatric surgery and how these changes lead to weight loss and a better metabolic profile. Microbiome analyses following *Roux-en-Y* gastric bypass (RYGB) or vertical banded gastroplasty have shown similar long-term effects on gut microbiota and fat loss [61]. In mice, RYGB has been shown to induce a rapid and maintained increase in the presence of *Gammaproteobacteria* (*Escherichia*) and *Verrucomicrobia* (*Akkermansia*) [62]. A study of microbiome modifications in 13 obese patients after bariatric surgery reported an increase in gut microbial diversity and identified alterations in the relative abundances of 31 species [63]. These changes in microbiota occurred in parallel with weight loss and functional and metabolic improvements: (i) an increased potential to assimilate multiple energy sources using transporters and phosphotransferase system, (ii) a better use of aerobic respiration, and (iii) the use of amino acids and fatty acids as energy sources.

Very recently, an investigation of a cohort of lean and obese individuals identified a distinct causal role of the gut microbiota in the development of obesity [64]. Liu et al. associated changes in microbiota composition of some obesity-related microbial species with circulating metabolites. In particular, the abundance of a glutamate-fermenting commensal species, *Bacteroides thetaiotaomicron*, was notably decreased in obese individuals and was inversely correlated with serum glutamate concentration. Furthermore, these authors reported that, after bariatric surgery, several microbial enzymatic functions become more similar to those of lean controls, including pathways involved in carbohydrate fermentation, citrate cycle, glycosaminoglycan degradation, and lipopolysaccharide (LPS) synthesis, as well as the production of aromatic amino acid and branched chain amino acids (BCAA), suggesting that weight-loss intervention by bariatric surgery reversed, at least partially, the reported obesity-associated microbial and metabolic alterations. Very recently, decreased gut microbial gene richness (MGR) has been associated with severe obesity [22] and, although bariatric surgery increased MGR one year after surgery, most RYGB patients remained with low MGR one year post-surgery, which emphasizes the need of additional strategies to improve the dysbiosis in severe obesity. Table 1 summarizes the changes in gut microbiota associated with obesity and microbiota-targeted interventions for this disease.

#### 2.1.4. Potential Gut Microbiota Biomarkers of Obesity

The use of metagenomics analyses to link obesity and the gut microbiota allows for the specific bacterial strains to be identified as potential biomarkers of obesity and of the development or progression obesity. Dominant bacterial phyla that are consistently identified in the guts of normal individuals include Firmicutes, Bacteroidetes, and Actinobacteria, with Verrucomicrobia and Proteobacteria being present at lower abundances [65,66]. It has been recently described that microbiota markers in adolescent and adult obese patients show different age-dependent traits [67]. In particular, *Faecalibacterium prausnitzii* and *Actinomyces* have been assigned to the microbiota of obese adolescents, whereas *Bacteroides caccae*, *Barnesiellaceae*, *Parabacteroides*, *Rikenellaceae*, and *Oscillospira* have been assigned to the microbiota of adolescents with normal weights. *F. prausnitzii* participates in the fermentation of non-absorbed carbohydrates, and its abundance in the guts of obese adolescents may contribute to increased energy recovery, leading to a higher dietary energy intake that may in turn contribute to the lower success of weight loss diets reported for individuals with a higher abundance of *F. prausnitzii* [68]. Other reports associate the butyrate production capacity of *F. prausnitzii* with a healthy state [69,70,71], an apparent contradiction that emphasizes the need of future studies using broader cohorts to identify distinct microbial biomarkers that can unequivocally associate obesity with a given microbiota profile.

#### 2.1.5. Clinical Trials

Of the 123 active clinical trials annotated to date in ClinicalTrials.gov (not finished or completed) that consider the gut microbiota as a therapeutic target in obesity, 55 (44.7%) employ dietary supplements or modifications in the diets of individuals in their interventions, 21 (17.07%) propose the use of probiotics, prebiotics, or synbiotics, 15 (12.2%) analyze the microbiota after bariatric surgery, 9 (7.3%) perform fecal transplantation interventions, 12 (9.7%) institute changes in patient lifestyle (ranging from exercise to mindfulness), 4 (3.2%) use approved drugs to adjust the gut microbiota, and 7 (5.7%) are strictly observational analyses. The important role of the gut microbiota in the alteration of the physiological systems involved in obesity is reflected by the wide variety of therapies based on counteracting their pathological modifications.

### 2.2. Inflammatory Bowel Disease

Crohn’s disease (CD) and ulcerative colitis (UC) are the primary pathological conditions associated with IBD and are described as a chronic and non-specific inflammation of the gastrointestinal tract [72]. There is some information regarding the correlation between changes in the gut microbiota and the development of IBD [73,74]. Regarding microbiota profiles, some studies have reported lower relative abundances of *Alistipes finegoldi* and *Alistipes putredinis*, as well as Firmicutes, Tenericutes, and Bacteroidetes in patients with IBD [75].

#### 2.2.1. Ulcerative Colitis

The gut microbiota associated UC is characterized by a high ratio of *B. fragilis*/*F. prausnitzii* and a low abundance of butyrate-producing bacteria (BPB) [76]. Some studies have described a decrease in bifidobacteria [73], *Akkermansia municiphila* [77], and *Clostridium* clusters IV, XIVa, and XVIII [78] in patients with UC compared with the control groups. Two recent studies have reported an increased diversity in *Clostridium* cluster XIVa in UC patients [77] as well as increases in the abundances of Bacteroidetes, *Bacilli*, Proteobacteria, and *Clostridium* clusters IX and XI [78]. Recently, Bacteroidetes has been reported to be absent in patients with active UC [79]. These conflicting results regarding the Bacteroidetes phylum might be explained by the different status of the UC disease.

FMT is currently being considered as a potential future therapeutic approach to restore normal intestinal microbiota. FMT-treated patients exhibit different outcomes with respect to their gut microbiota composition depending on whether they respond to the transplant. Thus, non-responders show significantly higher levels of Bacteroidetes versus responders [80,81]. In addition, patients in remission have augmented levels of *Barnesiella* spp., *Parabacteroides* spp., *Clostridium* cluster IV, and *Ruminococcus* species [82,83].

Interesting changes in intestinal microbiota have been described after pharmacological treatments. Patients treated with mesalazine exhibited decreases in the abundances in *Bifidobacterium* and *Lactobacillus* and increases in the abundances of *Klebsiella*, *Proteus*, *Citrobacter*, and hemolytic *Escherichia coli* [84]. After treatment with andecaliximab, the presence of *Clostridia* and *Akkermansia* was observed in patients with active UC [85]. Patients treated with vedolizumab and those with non-remission showed increased levels of *Streptococcus salivarius* [86], and corticosteroid treatment was observed to be related to an increase in the abundances of bifidobacteria and *Clostridium* and a decrease in that of *Faecalibacterium* [87].

Regarding the use of probiotics, Matsuoka et al. have described that the administration of *Bifidobacterium breve* Yakult to UC patients barely had an effect, with the only change being in the relative abundance of *Clostridium leptum*, which significantly increased [88]. However, Ananthakrishnan et al. have suggested that early clinical remission of UC patients could be predicted by microbial composition only at baseline with a weaker influence at the level of the species or genus [87].

As for the impact of diet on the gut microbiota of UC patients, a low fat diet has been reported to increase the presence of Bacteroidetes after four weeks of intervention [89]. 

#### 2.2.2. Crohn’s Disease

Recent investigations have described lower levels of *Clostridium coccoides*, *Clostridium leptum* and *Faecalibacterium prausnitzii* and a higher abundance of *Escherichia coli* in patients with CD [90].

A significant increase in *Escherichia coli* was reported in patients with CD subjected to FMT [91]. Another study investigating FMT in CD patients described decreases in the abundances of *Bacteroides*, *Roseburia*, *Phascolarctobacterium*, and *Eubacterium* along with increases in those of *Bilophila*, *Streptococcus*, *Clostridium*, and *Paraprevotella* [92].

Infliximab treatment has been reported to increase the abundance of *Clostridiales* in responders versus relapsing patients [93]. Similarly, ustekinumab has been shown to increase *Faecalibacterium* in responders compared with relapsing patients [94]. 

Diet is an important variable in the treatment of patients with CD. The administration of fermentable oligosaccharides, disaccharides, monosaccharides and polyols was observed to increase the levels of *Clostridium cluster* XIVa and *Akkermansia municiphila*, whereas that of *Faecalibacterium prausnitzii* was unaffected [95]. With the objective of evaluating differences in the microbial profiles associated with exclusive enteral nutrition and corticosteroid administration, the authors of one study observed a decrease in *Prevotella*, bifidobacteria, and *Enterobacteriaceae* in both assayed treatments [87]. Table 2 summarizes changes in gut microbiota associated with intestinal diseases and microbiota-targeted interventions for these diseases.

### 2.3. Non-Alcoholic Fatty Liver Disease

Non-alcoholic fatty liver disease (NAFLD) is characterized by an incremental increase in fat accumulation in the form of micro- and macrovacuoles of lipids in hepatocytes [96]. This disease is closely related to obesity and metabolic syndrome and exhibits features associated with abdominal obesity, insulin resistance, glucose intolerance, and T2D [97,98].

In addition to general overnutrition, specific diet factors, such as an elevated intake of sugar and/or fat, and genetic factors are crucial for its pathogenesis and progression [99]. Thus, an elevated body weight appears to not be the only risk factor associated with NAFLD [100], although alterations in gut microbiota have been reported as promoting the development of NAFLD by mediating inflammation, insulin resistance, bile acids, and choline metabolism [101]. Nevertheless, neither the pathophysiology of NAFLD nor the gut microbiota alterations in patients suffering for NAFLD have been completely characterized, and there are currently no effective drug therapies for NAFLD [102], with weight loss via diet and lifestyle modifications being the most successful care recommendations [103]. 

Host responses to changes in microbiota metabolism lead to an overwhelming presence of gut-derived microbial products and the activation of innate immunity and inflammation, resulting in the development of NAFLD. The presence of the dysbiotic microbiota and an altered intestinal barrier, possibly associated with disruption of tight-junctions (“leaky gut”), promotes the translocation of several bacterial products into the portal circulation. The interaction of bacterial products with toll-like receptors (TLRs) on the hepatic cell surface promotes the progression from simple steatosis to inflammation and fibrosis of the liver. For instance, the presence of *H. pylori* has been shown to induce gastric atrophy, with consequent acid losses predisposing small intestinal bacterial overgrowth, leaky gut, and portal endotoxin translocation [104]. 

Thus, interventions targeting the gut–liver axis through modulation of the gut microbiota by diet and/or pharmacological strategies appear to be a safe and sustainable tool to manage NAFLD. Manipulation of the gut microbiota has been primarily achieved through the use of probiotics, prebiotics, or symbiotic supplementation as well as through dietary intervention. For instance, very recently, Wang et al. reported that the alterations in the levels of bile acids (BAs) by gut bacteria through an isocaloric Mediterranean dietary intervention in 48 NAFLD patients did not alter fecal BA levels. Fecal BA levels and the microbiome do not appear to be responsible for the improvement in hepatic steatosis observed during dietary intervention in these patients [105]. In this respect and contrary to expectations, a study performed with 100 morbidly obese NAFLD patients undergoing laparoscopic sleeve gastrectomy surgery plus probiotic supplementation (Bio-25, Supherb, Israel) for six months showed higher abundances of Actinobacteria and *Collinsella* in the experimental group compared with the placebo. Although the alpha-diversity values never returned to the baseline levels after the intervention, an incremental improvement was observed at Month 6 compared with Month 0, and reductions were also observed at Month 12 vs. Month 6. However, the probiotic treatment did not have any added benefits beyond the influence of the surgery itself with respect to hepatic improvement, inflammatory or clinical outcomes [106]. Conversely, a prospective study that included 42 patients with NAFLD observed that those subjects receiving prebiotics supplements and lifestyle modification (i.e., diet and exercise) showed significant improvement in parameters related to liver function compared with those that only modified their lifestyle [102]. Similarly, prebiotic supplementation with inulin following metronidazole therapy plus a very low calorie diet was shown to ameliorate decreased liver function by reducing the levels of alanine aminotransferase in NAFLD subjects [107]. 

The potential benefits of probiotics administration have also been evaluated by Kobyliak et al., who reported that the co-administration of a probiotic with omega-3 in T2D patients with NAFLD improved serum lipid levels and metabolic profiles and reduced liver fat levels and the chronic systemic inflammatory state [108]. Along this line, the same group showed similar results after the administration of a concentrated biomass of 14 probiotic bacterial genera (including *Bifidobacterium*, *Lactobacillus*, *Lactococcus*, and *Propionibacterium*) [108]. However, in the abovementioned studies, details regarding the changes in gut microbiota associated with the probiotics/prebiotics supplementation were not described. In contrast, Ahn et al. [109] performed a study in which 68 NAFLD obese patients were randomly divided into prebiotic (receiving a mixture of probiotics *Lactobacillus acidophilus*, *Lactobacillus rhamnosus*, *Lactobacillus paracasei*, *Pediococcus pentosaceus*, *Bifidobacterium lactis*, and *Bifidobacterium breve*) or placebo groups. In the probiotics group, but not in the control group, a significant increase in all strains except *Lactobacillus paracasei* was observed as well as higher ratio Bacteroidetes/Firmicutes. Those patients that presented fatty liver improvement also showed increased abundances of *Ruminococcaceae-2*, *Lachnospiraceae-2*, *Coprococcus*, *Lachnospiraceae-1*, *Ruminococcus*, and *Dorea*. Additionally, patients with successful weight loss presented increases in the abundances of *Ruminococcaceae-2*, *Ruminococcaceae-1*, *Clostridiales-2*, *Lachnospiraceae-2*, and *Coprococcus*. All of these changes were associated with fatty liver improvements. 

The activation of inflammation due to both dysbiosis and alterations in intestinal permeability via TLRs signaling in hepatocytes induces progression from simple steatosis to non-alcoholic steato-hepatitis (NASH). This progression in dysbiotic NASH patients may be explained by the cytotoxicity associated with the increase in primary fecal BA, the primary/secondary fecal BA ratio, and plasma and hepatic BA concentrations [104]. Specifically, for NASH patients, therapies based on prebiotic supplementation consisting of the ingestion of oligofructose for 24 weeks improved liver histology independently of body weight. In addition, oligofructose increased the abundance of *Bifidobacterium*, the incremental increase in which inversely associated with obesity and plasma LPS [110]. Moreover, a 12-week treatment with a probiotic cocktail significantly altered the microbial structure in the feces of the experimental group, increasing towards the normal range without significantly altering the abundances of pathogenic enterobacteria and causing a significant decrease in liver inflammation without other adverse events [111].

Similarly, Alisi et al. reported changes in gut microbiota of obese children associated with the improvement of steatohepatitis and BMI after supplementation with VSL#3, a mixture of eight probiotic strains (*Streptococcus thermophilus*, bifidobacteria (*B. breve*, *B. infantis*, and *B. longum*), *Lactobacillus acidophilus*, *L. plantarum*, *L. paracasei*, and *L. delbrueckii* subsp. *bulgaricus*) for four months [112]. In contrast, synbiotic supplementation only had positive effects on anthropometric parameters associated with NASH (BMI, waist circumference, or uric acid levels) but not on gut permeability [113]. Most authors have concluded that modulation of gut microbiota brings added benefits by itself or in combination with other interventions (i.e., diet, exercise, or bariatric surgery) to NAFLD patients [106,108,114]. However, the heterogeneity of strategies (the use of pre- or probiotics, the length of treatment, the grade of steatosis, the presence of inflammation, etc.) makes it mandatory to understand and completely define what the precise changes causing the imbalance are and/or the changes in gut microbiota that lead from hepatic health to fatty liver disease. Along this line, evidence that the gut microbiota is altered in pediatric NAFLD and obese patients was investigated using targeted metagenomics and metabolomics (Table 3). The authors concluded that the combination of a low abundance of *Oscillospira* with high levels of 2-butanone may be a specific intestinal metagenomic and metabolomic profile for liver steatosis in children. Moreover, pediatric NASH patients presented high relative abundances of *Lachnospiraceae*, *Ruminococcus*, and *Dorea*. In addition, *Blautia* was extremely high in NASH but not in NAFLD or obese children, while *Oscillospira* was significantly less abundant in the NAFLD, NASH, and obese patients compared with controls, suggesting that changes in the gut microbiota are associated with the severity of fatty liver disease [45]. In this regard, differences in the microbiota of adult patients with biopsy-proven NAFLD, HCC, and fibrosis were also assessed. In this study, the composition of gut bacteria was determined by sequencing of 16S rRNA gene, the results of which showed decreased abundances of Bacteroidetes and *Faecalibacterium prausnitzii* in NAFLD patients compared with the healthy controls.

Furthermore, endotoxin levels were observed to be higher in NAFLD patients with severe fibrosis than in those with mild fibrosis, revealing that the mechanism of fibrotic progression via the endotoxin in NAFLD may be strongly associated with gut permeability. The latest results suggest that the distinct composition of the gut microbiota among NAFLD and HCC patients may offer a target for intervention or a marker for disease [115]. In addition, a recent study by Lelouvier et al. attempted to associate microbiota dysbiosis from blood and feces with LF and BMI > 40 kg/m^2^ patients in two different geographic populations. Only in one of the studied populations, specific bacterial taxonomic profiles and their functions were associated with LF. This finding suggests that there is a potential interaction between bacterial communities and an environmental factor specific to the geographical area in obese patients [116]. 

In summary, available studies use a large variety of interventions (probiotic, prebiotics, symbiotic supplementation, diet, or other approaches) and different patient baselines and treatment times (from weeks to months), making it difficult to draw clear conclusions and highlighting the need for further research in this field. From our point of view, the analysis of the gut microbiota under unhealthy conditions remains to be defined, as well as how it is affected by diet, BMI, or age as well as others factors, such as the grade of steatosis and geographical environment.

### 2.4. Insulin Resistance Syndrome

Insulin resistance syndrome (IRS) or metabolic syndrome is characterized by hyperinsulinemia and an increased prevalence of obesity, hypertension, dyslipidemia, and T2D [117]. NAFLD is the hepatic component of IRS [97]. 

IRS is currently a global burden, with 20–25% of the adult population worldwide suffering from this disease [118]. In addition to genetic, lifestyle, and nutritional factors [119,120,121], alterations in gut microbiota (dysbiosis) are also associated with IRS status [118,122,123,124]. The primary contributors to the development of IRS are as follows: LPS from the gut microbiota, which can induce a chronic subclinical inflammatory process through activation of TLR4; a reduction in circulating SCFA; alterations in the secretion of glucagon-like peptide-1 (GLP-1) mediated by bile acids; and an increased circulation of branched-chain amino acids (BCAA) [125]. 

The results of a metagenomic analysis performed with 58 IRS patients from Kazakhstan revealed differences in gut microbiota composition of these patients compared with the healthy controls (e.g., significant reductions in the Firmicutes/Bacteroidetes ratio, bifidobacteria, and *Subdoligranulum* and increased *Prevotella*). The results of this study disagree with previously published studies that were primarily performed with European subjects in whom an increased Firmicutes/Bacteroidetes ratio was observed, a discrepancy that is probably due to the dietary, lifestyle, and geographical features of the Kazakh population [126].

With the purpose of reversing the different IRS-associated changes that occur in the gut microbiota and reduce the impact of this disease, different approaches are being investigated to increase the therapeutic strategies available to combat this syndrome (Table 4). Among these strategies are dietary changes, nutritional supplementation, and the administration of prebiotics, probiotics, FMT [81,127,128,129,130,131,132,133], and antibiotics [134]. Haro et al. evaluated the consumption of both a Mediterranean and a low-fat diet in 138 IRS patients for two years. Firstly, they observed higher abundances of the genera *Bacteroides*, *Eubacterium,* and *Lactobacillus* and reduced abundances of *B. fragilis* group members, *P. distasonis*, *B. thetaiotaomicron*, *F. prausnitzii*, *F. nucleatum*, *B. longum*, *B. adolescentis*, the *R. flavefaciens* subgroup, and *E. rectale* in IRS patients at time 0. After the intervention, only the Mediterranean diet had induced significant changes (increased abundances of *P. distasonis*, *B. thetaiotaomicron*, *F. prausnitzii*, *B. adolescentis,* and *B. longum*) in IRS patients, suggesting that, although IRS persisted after the intervention, a Mediterranean diet may contribute to partially restoring the beneficial components of the gut microbiota [135]. These authors also performed a similar study (an evaluation of the effect of Mediterranean and low-fat diet for two years) in 33 obese patients with severe IRS versus 32 obese patients that did not fulfill IRS criteria and 41 normal-weight subjects. In this study, they observed a significant decrease in the Firmicutes/Bacteroidetes ratio after the consumption of the low-fat diet, but only a decreasing trend for the Mediterranean diet. Additionally, the abundances of the genera *Bacteroides*, *Prevotella*, and *Faecalibacterium* increased in IRS patients for both diets, Mediterranean and low-fat, and reduced abundances of the genera *Streptococcus* and *Clostridium* were also observed in the low-fat diet group. In contrast, the group that received the Mediterranean diet did not exhibit changes in the abundance of these genera but did exhibit increases in those of the genera *Roseburia* and *Ruminococcus* and in the bacterial species *P. distasonis* and *F. prausnitzii*. Regarding the effect of these diets on metabolic parameters, the triglyceride levels decreased in the IRS group and remained unchanged in the other groups. These results suggest that the chronic intake of Mediterranean or low-fat diets partially ameliorates the gut microbiome dysbiosis in severe IRS patients but that this improvement depends on the degree of the metabolic dysfunction [136]. 

Salonen et al. reported on changes in the gut microbiota of 14 IRS patients fed four different diets in a sequential fashion for 10 weeks as follows: (1) a standard diet for weight maintenance (M) for one week; (2) a diet with a high content of type 3 resistant starch (RS) for three weeks; (3) a diet with a high content of non-starch polysaccharides (NSP) for three weeks; and, (4) a weight loss diet with high protein and medium carbohydrate levels (WL) for another two weeks. The main results after the different diet interventions were as follows: the RS diet increased *Ruminococcus*-related bacterial phylotypes and the microbiota diversity, the NSP diet primarily led to increases in the abundance of *Lachnospiraceae*, and the WL diet reduced the bifidobacteria. Notably, the dietary responsiveness of the microbiota varied substantially among patients and was inversely associated with their diversity. This result suggests that individuals can be stratified into responders and non-responders based on the features of their intestinal microbiota. A positive correlation between fecal bifidobacteria and plasma insulin was also detected in IRS patients [137].

Another strategy to modulate the gut microbiota composition through nutritional supplementation in IRS was described in a study by Moreno-Indias et al. performed with 10 IRS patients. The intake of two red wines of equal composition except for the ethanol content (regular and ethanol-free red wines) for 30 days significantly increased the number of fecal bifidobacteria, *Lactobacillus* and butyrate-producing bacteria (*Faecalibacterium prausnitzii* and *Roseburia*) at the expense of less desirable groups of bacteria, such as LPS producers (*Escherichia coli and Enterobacter cloacae*). These changes were attributed to the polyphenols present in both wines, and were associated with a reduction in IRS risk markers, triglycerides, HDL-cholesterol, glucose, and glutamic pyruvic transaminase [138]. A study by Ni et al. observed higher proportions of *Lactobacillus* and *Bifidobacterium* and reduced abundances of *Anaerostipes*, *Coprococcus*, and *Ruminococcus* in 12 elderly IRS patients compared with healthy individuals of the same age. A four-week treatment with Yangyin Tiluo Decoction (a Chinese Herbal Formula) decreased the abundance of the order *Bacteroidales*. Lipoprotein(a) levels were also reduced in plasma in correlation with the abundance of *Acinetobacter* species [139]. 

Whole grain has been described to increase the abundances of *Faecalibacterium prausnitzii*, *Prevotella copri*, and *Clostridiales* and decrease those of *Bacteroides thetaiotaomicron* compared with a refined grain diet. Whole grain ingestion reduced body weight and systemic low-grade inflammation but did not alter insulin sensitivity [140].

With respect to interventions with prebiotics and probiotics, the intake of barley β-glucans for four weeks by 27 IRS patients induced an increase in the abundance of *Agathobacter rectalis* and decreased that of *Coriobacteriales* and *Clostridiales* and was associated with a reduction in total plasma cholesterol [141]. In a 12-week study, Stadlbauer et al. evaluated the effect of *Lactobacillus casei* Shirota in 28 IRS patients (13 patients took β-glucans and 15 did not). Despite inducing an increase in the abundances of *Parabacteroides* and *Lactobacillus casei* Shirota, the supplementation did not restore gut microbiota composition, the gut barrier or the Bacteroidetes/Firmicutes ratio [142].

Smits et al. tested an FMT-based strategy in 20 patients with IRS [134], where patients received feces from vegan donors and another 10 patients received their own feces. Feces from vegans induced subtle changes, such as increased abundances of several microbial groups belonging to the *Lachnospiraceae* family, including bacteria related to *Bryantella formatexigens* and *Megamonas hypermegale* as well as *L. bovis* two weeks after transplantation. However, FMT did not induce any improvement in parameters related to vascular inflammation. Furthermore, no changes occurred in the group of patients that was subjected to autologous transplantation [134]. 

With regard to the use of antibiotics, the effect of oral vancomycin treatment (500 mg for seven days) on the gut microbiota in 10 IRS patients was described by Vrieze et al. [143]. The primary findings of this study were a reduction in fecal microbial diversity, a decrease in Gram-positive bacteria (especially Firmicutes) with a concomitant increment in Gram-negative bacteria (especially Proteobacteria). Vancomycin also decreased the levels of fecal secondary bile acids with a simultaneous postprandial increase in primary circulating bile acids, which was associated with an altered abundance of Firmicutes. With respect to metabolic effects, the administration of vancomycin reduced peripheral insulin sensitivity [143].

Overall, findings supporting the beneficial effects of various interventions (diet, prebiotics, probiotics, FMT, etc.) on gut microbiota as a strategy to treat IRS appear to be scarce and modest. In addition, the persistence of these changes remains to be evaluated. In our view, both the number of studies and enrolled patients are small, and the inter-individual variation between patients should also be considered. Consequently, further research in this area of study is required to address the aforementioned weaknesses.

### 2.5. Diabetes Mellitus Type II

Over 60 million people are diagnosed with T2D in Europe [144]. According to WHO, diabetes will be the seventh leading cause of death in 2030 [145]. Factors such as a healthy diet, regular physical activity, and body weight maintenance at normal levels prevent T2D development, delay its progression, and help mitigate its pathological consequences. Prediabetes and high blood glucose levels maintained over time without symptomatology are associated with overweight and obesity. Because prediabetes leads to the development of diabetes in 70% of cases, a prevention strategy is highly recommended in this state. T2D could be linked to the gut microbiota composition, and it is directly responsible for the induction of a low-grade inflammatory state. The composition of the gut microbiota also plays a significant role in the development of pre-diabetes conditions such as resistance to insulin. The main alterations in gut microbiota associated with T2D have a significantly lower prevalence of Firmicutes and enrichment of Bacteroidetes and Proteobacteria [140].

Growing evidence indicates that functional foods, such as prebiotics, probiotics, and postbiotics, may be used in the prevention and treatment of diabetes [146,147,148]. Table 5 summarizes microbiota-targeted interventions for T2D.

Totum63 is an association of plant principles for the treatment of T2D and prediabetes. In db/db mice, a well-established obesity-induced animal model of TD2 in which mice carry a mutation in the leptin receptor gene, Totum63 delayed the defect in insulin secretion, suggesting a protective role on pancreatic beta-cells. Furthermore, in a phase I/II clinical trial Totum63 administration also induced improvements in glucose levels and insulin response to a standardized breakfast in healthy participants. Moreover, Totum63 treatment has been described to prevent the dysbiosis induced by a high fat diet [149].

Metformin is the most frequent treatment used for T2D, and this drug has been reported to significantly decrease the abundance of opportunistic bacteria [150] and significantly increase that of beneficial bacteria, particularly *Blautia* spp. Moreover, a Chinese herbal formula has been reported to increase *Blautia* and *Faecalibacterium* abundances in an open label study in T2D patients. Both treatments were linked with diabetes improvements with respect to glucose levels, a homeostasis model assessment of insulin resistance (HOMA-IR), and lipid homeostasis [151]. 

Canfora et al. studied the effects of GOS in 44 obese/overweight pediatric individuals [152]. The supplementation of diets with GOS increased the abundance of *Bifidobacterium* species in feces, while the microbial richness and diversity in fecal samples was unaffected. No differences in fecal or fasting plasma SCFA concentrations or in the systemic concentrations of gut-derived hormones, incretins, LPS-binding protein, or other markers of inflammation were observed. In addition, no significant alterations in peripheral and adipose tissue insulin sensitivity, body composition, or energy and substrate metabolism were observed [152].

The microbiome may also be involved in determining how glycemic control impacts weight change for different diets. The administration of SCFAs has been reported to result in a wide range of health benefits, including improvements in blood lipid profiles, glucose homeostasis, and body composition as well as in reduced body weight [148]. High butyrate levels were observed to be associated with beneficial effects on insulin sensitivity and glucose levels [153,154]. A randomized, double-blind study was carried out with 81 T2D patients divided into two three-month treatment groups one group took a placebo, while the other group followed a reduced-energy diet comprising high-fiber, polyphenol-rich, and vegetable-protein functional foods. Intervention with the last diet significantly modified fecal microbiota compared with the placebo, increasing the abundances of *F. prausnitzii* and *A. muciniphila* and decreasing that of *P. copri*.

SCFAs are produced by various human gut microbes. SCFAs act as an energy source to the colonic epithelium and are also sensed by host signaling pathways that modulate appetite and inflammation. A deficiency in gut SCFAs is associated with type 2 diabetes. Zhao et al. observed that a high-fiber diet promoted the growth of SCFA-producing organisms in diabetic humans [147,155]. Furthermore, the high-fiber diet induced changes in the entire gut microbe community and correlated with elevated levels of glucagon-like peptide-1, a decrease in acetylated hemoglobin levels, and improved blood–glucose regulation [147,155]. 

In a clinical trial with T2D patients, the relative abundance of *A. muciniphila*, a mucin-associated bacterium with anti-inflammatory properties, increased after inulin and sodium butyrate prebiotic supplementation [156,157,158]. 

A type of black tea from China contains some polysaccharides and polyphenols that improve glucose and lipid metabolism in prediabetic subjects by modifying the gut microbiota (decreasing *Faecalibacterium* and *Oscillospira*). In particular, GLP-1 and GLP-2 serum levels increased after 12 weeks in the group of patients that drank the tea compared with those who received a placebo [159].

Another dietary resistant starch-based prebiotic has been shown to positively affect metabolic health markers. In humans, resistant starch fermentation may enhance the abundances of specific gut microbiota taxa, such as *Bifidobacterium*, *Ruminococcus bromii*, and *Eubacterium rectale*, with associated improvements in insulin response, body fat reserve, and cholesterol levels [160]. Transglucosidase (TGD) produces oligosaccharides from starch in the human intestinal tract. Shimozato et al. observed that TGD administration to patients with T2D for 12 weeks significantly affected the fecal microbiota content, increasing *Clostridium* cluster IV, *Clostridium* subcluster XIVa, *Clostridium* cluster XVIII, and fecal pH and decreasing the order *Lactobacillales* in patients with bowel movement disorder compared with the controls. The TGD treatment significantly improved bowel movements compared with the placebo treatment, and this effect was not observed in patients without bowel movement disorder [161].

Several lines of clinical evidence suggest that the use of probiotics as alternative therapeutics against T2D increases the quality of life [146,162,163,164]. Some clinical trials were reviewed to confirm the beneficial effects and deepen understanding of the underlying mechanisms [165], resulting in a combination of life-style intervention and probiotic supplementation in prediabetic adolescents being proposed [163]. The efficacy of probiotics appears to depend on the bacterial strain and the study outcome. *Lactobacillus casei* strain *Shirota*-fermented milk was investigated in T2D pathologies, the results of which confirmed that this probiotic reduced bacterial translocation in Japanese individuals with T2D [146]. Furthermore, the administration of *Lactobacillus reuteri* to T2D patients has been shown to improve insulin sensitivity [162] (Table 5). As for postbiotics, defined as secreted factors, cellular components and metabolites of bacteria that, upon delivery to a host, may exert biological effects on it. Microbial components from live or dead bacteria can also be considered a source of postbiotics. Examples of postbiotics such as microbial-derived short-chain fatty acids or flavonoids may directly influence host feeding behavior, energy metabolism, insulin secretion, and insulin sensitivity. These postbiotic properties should be considered in obesity-related metabolic disease and represent a promising alternative to current treatments [166].

**Figure 1:**
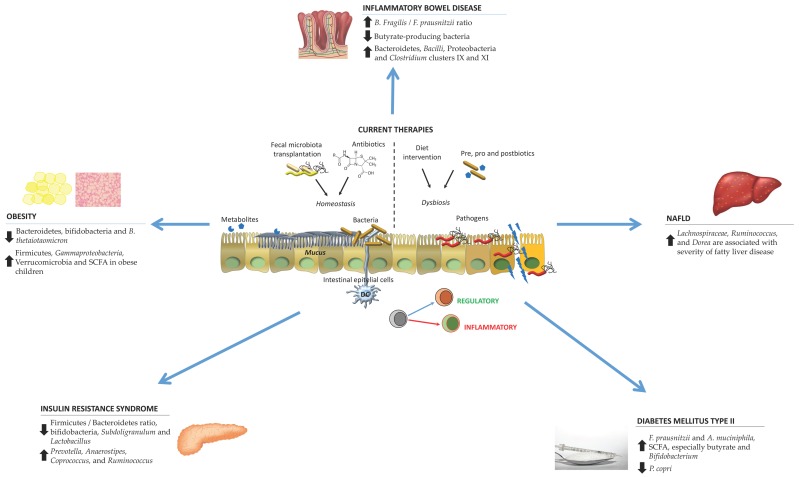
Schematic representation of the microbiota alterations in various disorders and the current therapies to counteract their effects. Abbreviations. DC: dendritic cells; NAFLD: non-alcoholic fatty liver disease; SCFA: short-chain fatty acids.

Figure 1 summarizes the microbiota alterations in particular pathologies as well as current therapies.

## 3. Future Directions

Maintaining a healthy gut ecosystem is essential, and alterations in the diversity, quantity, and stability of a healthy microbiota has been associated with obesity, IBD, and IRS as well as other metabolic disorders such as NAFLD and T2D. Since the prevalence of these diseases continues to rise worldwide, the modulation of the gut microbiota through various interventions (e.g., prebiotics, probiotics, and FMT) is a research area of increasing interest given their potential as tools to prevent or treat these diseases. However, conflicting results and limited efficacy have been reported to date. The different durations and doses of the interventions, as well as the technology used for sequencing the microbiome makes comparisons among studies difficult. 16S approaches for microbiome analyses are well suited for analysis of a large number of samples, i.e., multiple patients, longitudinal studies, and so on. However, this approach offers limited taxonomical and functional resolution. Moreover, it should be pointed out that using primers for different regions of the 16S rRNA gene may lead to discordant results due not only to the distinct binding affinities for the corresponding flanking conserved regions, but also due to the resolution of each variable region across taxa [167]. In contrast, shotgun metagenomics is usually more expensive but offers increased resolution, enabling a more specific taxonomic and functional classification of sequences, as well as the discovery of new bacterial genes and genomes [168].

FMT, which has yielded promising results for treating certain disorders, such as *Clostridium difficile* infections, is a clear example of this issue and should be addressed in future studies. Stool donor preparation and the transplantation method (endoscopy, nasointestinal tubes, or capsules) must be standardized, and the long-term potential adverse effects should be studied in greater detail.

Among the next-generation beneficial microbes that have been identified, *Akkermansia muciniphila* and *Faecalibacterium prausnitzii* are promising candidates. *A. muciniphila* has been reported to be inversely associated with obesity, diabetes, cardiometabolic diseases, and low-grade inflammation. Besides the numerous correlations observed, a large body of evidence has demonstrated the causal beneficial impact of this bacterium in a variety of preclinical models [169]. Many studies have shown that *F. prausnitzii* abundance is reduced in different intestinal disorders. It has been proposed that *F. prausnitzii* monitoring may therefore serve as a biomarker to assist in gut diseases diagnostics [170]. Testing these strategies in many more clinical trials and with great numbers of human subjects is clearly needed.

## Figures and Tables

**Table 1:** Microbiota changes associated with obesity.

Reference	Characteristics	Disease	Method	Primary Results
Turnbaugh et al., 2009 [34]	154 adult female monozygotic and dizygotic twin pairs concordant for leanness or obesity	Obesity	Sequencing (16S rRNA)	Gut microbiomes are shared among family members, but the gut microbial community varies in each individual.
Ignacio et al., 2016 [40]	Correlation between BMI and fecal microbiota in 84 children	Obesity	qRT-PCR	Significant association between the number of *Lactobacillus* spp. and *B. fragilis* group members and BMI.
Riva et al., 2017 [44]	Characterization of the gut microbiota in 78 obese and normal-weight children aged 6 to 16	Obesity	Sequencing (16S rRNA)	Elevated levels of Firmicutes and depleted levels of Bacteroidetes.
Nicolucci et al., 2017 [48]	42 obese children who received either oligofructose-enriched inulin or placebo	Obesity	Sequencing (16S rRNA)	Significant increases in species of the genus *Bifidobacterium* and decreases in *B. vulgatus* within the group that consumed oligofructose-enriched inulin.
Zhang et al., 2015 [54]	Intervention trial in 38 Prader-Willi syndrome and simple obesity children.	Prader-Willi syndrome and obesity	Analysis of prevalent bacterial draft genomes assembled directly from metagenomic datasets	Non-digestible carbohydrates induced significant weight loss and concomitant structural changes in the gut microbiota.
Bai et al., 2018 [55]	267 children (7–18 years old) analyzed according to their lifestyles	Obesity	Sequencing (16S rRNA)	Lower BMI and exercise frequency were associated with depleted Actinobacteria; Proteobacteria was significantly enriched in individuals with higher BMI levels; and Firmicutes was significantly enriched in individuals participating in frequent exercise.
Rampelli et al., 2018 [56]	70 children analyzed in a two-time point 4-year prospective study	Pre-obese	Sequencing (16S rRNA)	Pre-obese dysbiosis and unhealthy diets were correlated and suggested to be predictors of obesity.
Tremaroli et al., 2015 [61]	Gut microbiome analysis of 14 women 9.4 years after bariatric surgery was performed	Obesity	High-quality Illumina reads alignment analysis	Bariatric surgery induces long-term alterations in the human gut microbiome. Surgically altered microbiomes contribute to fat mass regulation.
Palleja et al., 2016 [63]	Gut microbiome analysis 1 and 3 months after bariatric surgery in 13 patients	Obesity	Shotgun metagenomic sequencing	31 microbial species showed altered relative abundances within the first 3 months, 16 of which maintained their altered relative abundances 1 year after surgery. *F. prausnitzii* was the only species that decreased in relative abundance.
Liu et al., 2017 [64]	Gut microbiome analysis of obese and post-bariatric intervention individuals in a cohort of 257 lean and obese young individuals	Obesity	Metagenome-wide association	Abundance of *B. thetaiotaomicron* was markedly decreased in obese individuals. Bariatric surgery intervention reversed obesity associated microbial alterations, including the decreased abundance of *B. thetaiotaomicron*.
Aron-Wisnewsky et al. [22]	61 severely obese subjects of whom 24 were followed 1, 3, and 12 months post-bariatric surgery	Obesity	Shotgun metagenomics	Although bariatric surgery increased MGR one year after surgery, most RYGB patients remained with low MGR one year postsurgery.
Del Chierico et al., 2018 [67]	Gut microbiome analysis of 69 adolescent and adult patients	Obesity	Sequencing (16S rRNA)	Microbial markers, *F. prausnitzii* and *Actinomyces* assigned to the microbiota of obese adolescents. *Parabacteroides*, *Rikenellaceae*, *Bacteroides caccae*, *Barnesiellaceae* and *Oscillospira* were assigned to the microbiota of normal weight adolescents.
Le Chatelier et al., 2013 [68]	Gut microbiome analysis of 292 adult patients	Obesity	Sequencing (16S rRNA)	Individuals with low bacterial richness are characterized by increased overall adiposity compared to high bacterial richness individuals.

**Abbreviations:** BMI: body mass-index; MGR: microbial gene richness; qRT-PCR: quantitative polymerase chain reaction; rRNA: ribosomal ribonucleic acid; RYGB: *Roux-en-Y* gastric bypass.

**Table 2:** Microbiota changes associated with inflammatory bowel disease.

Reference	Disease	Intervention	Primary Results	Method
Sitkin et al., 2018 [76]	40 UC patients	-	High *B. fragilis/F. prausnitzii*, depleted BPB, low *Bifidobacterium.*	qRT-PCR
Ishikawa et al., 2018 [81]	36 UC mild–severe patients	FMT+AFM pretreatment	Bacteroidetes were recovered.	Sequencing (16S rRNA)
Matsuoka et al., 2018 [88]	43 UC remission patients, 20–70 y/o	*Bifidobacterium breve* Yakult	Increase in *C. leptum.*	qRT-PCR
Phillips et al., 2018 [89]	UC quiescent	Low fat diet	High Bacteroidetes.	-
Ananthakrishnan et al., 2017 [86]	43 UC patients	Vedolizumab	In non-remission, high *S. salivarius.*	Sequencing (V4 16S rRNA)
Lamere et al. 2017 [85]	59 UC patients	Andecaliximab	High *Clostridia* and *Akkermansia.*	Sequencing
Fuentes et al., 2017 [80]	33 UC mild–moderate patients	FMT	Low *Clostridium* cluster XIVa, non-responders had high Bacteroidetes.	Sequencing (16S rRNA)
Dobrolyubova et al., 2017 [84]	162 UC patients, 35–41 y/o	5-ASA	In remission, low *Bifidobacilles* and *Lactobacillus*, high *Klebsiella*, *Proteus*, *Citrobacter*, and hemolytic *E. coli.*	-
Lee et al., 2016 [79]	22 UC active and remission patients, >18 y/o	-	Bacteroidetes absent in patients with active UC.	Sequencing (16S rRNA)
De Caro et al., 2016 [73]	14 UC active and remission patients, mean 39 y/o	Infliximab, adalimumab, azathioprine or 5-ASA	Low bifidobacteria.	Metagenomic
Paramsothy et al., 2016 [82]	81 UC patients	FMT	*Barnesiella* was associated with remission; *Fusobacterium* and *Sutterella* were associated with a lack of remission.	Sequencing (16S rRNA)
Hart et al., 2016 [87]	7 UC patients, 5–18 y/o	CS	High bifidobacteria and *Clostridium* and low *Faecalibacterium.*	Sequencing (16S rRNA)
Rossen et al., 2015 [78]	58 mild–moderate UC patients	-	Low *Clostridium* clusters IV, XIVa and XVIII and high in Bacteroidetes, *Bacilli*, Proteobacteria and *Clostridium* cluster IX and XI.	qRT-PCR
James et al., 2014 [77]	37 UC patients, >18 y/o	-	UC patients have more *Clostridium* cluster XIVa. Lower *A. muciniphila.*	qRT-PCR
Doherty et al., 2017 [94]	350 moderate–severe CD patients, 18–76 y/o	Ustekinumab	High *Faecalibacterium* in responders and remission patients.	Sequencing (V4 16S rRNA)
Zhou et al., 2017 [93]	16 CD patients	Infliximab	Incremental change in *Clostridiales*.	Sequencing (V4 16S rRNA)
Yang et al., 2017 [92]	31 active CD patients	FMT	Low *Bacteroides*, *Roseburia*, and *Phascolarctobacterium*, *Eubacterium;* high *Bilophila*, *Streptococcus*, *Clostridium* and *Paraprevotella.*	Sequencing (V4 16S rRNA)
Hart et al., 2016 [87]	22 CD patients, 5–18 y/o	EEN or CS	Decreased in *Prevotella*, *Bifidobacteria* and *Enterobacteriaceae*.	Sequencing (16S rRNA)
Halmos et al., 2015 [95]	8 quiescent CD patients	FODMAP diet	Increased *Clostridium* cluster XIVa and *A. muciniphila. F. prausnitzii* was unaltered.	-
Suskind et al., 2015 [74]	9 mild–moderate CD patients, 12–19 y/o	FMT	High *E. coli* in response to inflammation.	Sequencing (V4 16S rRNA)
Rajca et al., 2015 [90]	19 relapser and 14 non-relapser patients	-	Low *C. coccoides*, *C. leptum* and *F. prausnitzii;* high *E. coli.*	Sequencing (V4 16S rRNA)

**Abbreviations:** 5-ASA: mesalazine; AFM: amoxicillin-fosfomycin-metronidazole; BPB: butyrate-producing bacteria; CD: Crohn’s disease; CS: corticosteroid; EEN: exclusive enteral nutrition; FMT: fecal microbiota transplantation; FODMAP: fermentable oligo-di-mono-saccharides and polyols; IBD: inflammatory bowel disease; qRT-PCR; quantitative polymerase chain reaction; rRNA: ribosomal ribonucleic acid; UC: ulcerative colitis; V4: hypervariable 16S region; y/o: years old.

**Table 3:** Microbiota changes associated with non-alcoholic fatty liver disease.

Reference	Characteristics	Intervention	Time (weeks)	Methodological Procedure	Primary Results
Del Chierico et al., 2017 [45]	61 children and adolescents (7–16 y/o). NAFLD (*n* = 27), NASH (*n* = 26), or obesity (*n* = 58)	NA	NA	Metagenomics and metabolomics analyses	Firmicutes, Bacteroidetes, Proteobacteria, and Actinobacteria were the principal differences.
Kessouku et al., 2017 [115]	201 adults. NAFLD = 143 (77 mild fibrosis and 56 severe fibrosis)	NA	NA	16S rRNA gene sequencing and blood endotoxin activity assay	*F. prausnitzii* decreased in NAFLD patients and elevated blood-endotoxin in NAFLD.
Lelouvier et al. 2016 [116]	44 adults (40–60 y/o) BMI > 40. Fibrosis 71 blood and fecal sample from Italy, 37 blood and 44 fecal samples from Spain	NA	NA	16S rRNA gene quantitation by qRT-PCR and 16S metagenomic sequencing	Changes in *Sphingomonas* and *Bosea* correlated significantly with fibrosis. *Ruminococcaceae*, *Lachnospiraceae*, *Coriobacteriaceae*, and *Fusobacteriaceae* are modified in LF.
Anh et al., 2018 [109]	34 adults Obesity plus NAFLD	Mixture of lactobacilli and bifidobacteria	12	qRT-PCR and 16S rRNA gene microbiome sequencing	Fatty liver improvement related to increases in *Ruminococcaceae-2*, *Lachnospiraceae-2*, *Coprococcus*, *Lachnospiraceae-1*, *Ruminococcus*, and *Dorea.*
Bomhof et al., 2018 [110]	Adults NASH	Oligofructose	24	Not indicated	Increase in *Bifidobacterium*. Decreased *Clostridium* clusters XI and I from prebiotic supplementation in patients with NASH.
Manzhalii et al., 2017 [111]	38 adults NASH	Probiotic cocktail: *lactobacilli*, bifidobacteria, and *S. thermophilus*	12	Not indicated	Increased abundances of bifidobacteria, *Lactobacillus*, *E. coli*, and *E. faecalis*, among others.

**Abbreviations:** BMI: body mass index; LF: liver fibrosis; NA: non-applicable; NAFLD: non-alcoholic fatty liver disease; NASH: non-alcoholic steatohepatitis; RT-qPCR: quantitative polymerase chain reaction; rRNA: ribosomal ribonucleic acid; y/o: years old.

**Table 4:** Microbiota changes associated with insulin resistance syndrome.

Reference	Characteristics	Procedure	Main Results
Kushugulova et al., 2018 [126]	58 IRS patients	16S rRNA gene sequencing	IRS patient showed reduced Firmicutes/Bacteroidetes ratio, *Bifidobacteria* and *Subdoligranulum* and increased *Prevotella.*
Haro et al., 2017 [136]	33 adult obese patients with severe IRS vs. 32 non IRS obese patients and 41 normal-weight subjects	16S rRNA gene sequencing	After administration of MD and LF diet for 2 years, MD decreased the F/B ratio, Bacteroidetes, *Bacteroides* and *Prevotella*, and increased *Faecalibacterium* in the IRS group.
Haro et al., 2016	138 IRS patients	16S rRNA gene sequencing	At time 0, increased *Bacteroides*, *Eubacterium.* and *Lactobacillus* genera and reduced the *fragilis* group, *P. distasonis*, *B. thetaiotaomicron*, *F. prausnitzii*, *F. nucleatum*, *B. longum*, *B. adolescentis*, the *R. flavefaciens* subgroup, and *E. rectale* in IRS patients at time 0. In a two-year intervention, Mediterranean diet and low-fat high-carbohydrate diet partly restored *P. distasonis*, *F. prausnitzii*, *B. thetaiotaomicron*, *B. adolescentis*, and *B. longum* levels.
Salonen et al., 2014 [137]	12 adult IRS patients	HITChip phyloge Neticmicroarray and q-PCR	Dietary intervention: 1 week M diet, 3 weeks RS diet, 3 weeks NSP diet, and 3 weeks WL diet. Multiple *Ruminococcaceae* phylotypes increased with the RS diet, and *Lachnospiraceae* phylotypes were primarily increased by the NSP diet.
Moreno-Indias et al., 2015 [138]	10 adult IRS patients	RT-PCR	Intake of wine and de-alcoholized red wine for 30 days/each increased bifidobacteria and *Lactobacillus* and *Faecalibacterium prausnitzii* and *Roseburia* and decreased *Escherichia coli* and *Enterobacter cloacae.*
Ni Y el al., 2018 [139]	12 elderly IRS patients (60–90 y/o)	16S rRNA gene sequencing	YDT supplementation for 4 days reduced *Bacteroidales Incertae Sedi*, *Enterobacteriaceae Incertae Sedis* and circulating lipoprotein(a) in correlation with *Acinetobacter* species.
Roager et al., 2019 [140]	50 IRS patients	16S rRNA gene sequencing	Administration of whole vs. refined grains for 8 weeks increased *F. prausnitzii*, *P. copri,* and *Clostridiales* but decreased *B. thetaiotaomicron.*
Smits et al., 2018 [134]	10 adults IRS	16S rRNA gene sequencing	Vegan FMT increased the levels of *Lachnospiraceae*, especially *B. formatexigens* and *M. hypermegale*, as well as *L. bovis.*
Velikonja et al., 2018 [141]	27 adults IRS patients	qRT-PCR, and 16S rRNA gene sequencing	β-Glucans induced an increase in *A. rectalis* and decreased the levels of *Coriobacteriales* and *Clostridiales* associated with a reduction in total plasma cholesterol.
Stadlbauer et al., 2015 [142]	13 adults IRS patients	16S rRNA gene sequencing	Intake of LcS for 12 weeks increased *Parabacteroides* but did not restore the gut microbiota composition, gut barrier, or Bacteroidetes/Firmicutes ratio.
Vrieze et al., 2014 [143]	100 adults IRS patients	qRT-PCR and Human Intestinal Tract Chip microarray	Administration of 500 mg/day of vancomycin for 1 week reduced Gram-positive bacteria (especially Firmicutes), secondary bile acids and peripheral insulin sensitivity while increasing Gram-negative bacteria (especially Proteobacteria) and reducing peripheral insulin sensitivity.

**Abbreviations:** F/B: Firmicutes/Bacteroidetes ratio; FMT: fecal microbiota transplantation; IRS: insulin resistance syndrome; *LcS*: *Lactobacillus* *casei* Shirota; M: standard diet at weight maintenance; MD: Mediterranean diet; NSP: high in non-starch polysaccharides; qRT-PCR; quantitative polymerase chain reaction; rRNA: ribosomal ribonucleic acid; RS: one high in type 3 resistant starch; V4: hypervariable 16S region; YDT: Yangyin Tiluo Decoction; y/o: years old.

**Table 5:** Microbiota changes associated with diabetes mellitus type II.

Reference	Characteristics	Procedure	Primary Results
Stefanaki et al., 2018 [163]	RCT with 50 adolescents. Probiotics and healthier lifestyle interventions	Body composition, glycemic and gut microbiota measurements	Probiotic administration was safe and useful for preventing the onset of pre-diabetes.
Tong et al., 2018 [151]	RCT in T2D and hyperlipidemia patients for 12 weeks with metformin and Chinese medicine treatment in 450 patients	16S rRNA gene (V3 and V4 regions) sequencing	Significantly decreased hyperglycemia and hyperlipidemia, enrichment in *Blautia* and *Faecalibacterium* spp.
Zhao et al., 2018 [155]	43 Chinese patients administered a high-fiber diet/prebiotics and a control. Both groups were treated with acarbose	Identification of SCFA-producing bacterial strains by metagenomic sequencing	Increased SCFA levels in the human bowel of the dietary fibers/prebiotics group. Improvement in hemoglobin A1c levels by elevating glucagon-like petide-1 production.
Roshanravan et al., 2018 [164]	59 overweight and obese patients with T2D received sodium butyrate, inulin powder or both or a placebo	16S rRNA gene analysis of *A. muciniphila* by quantitative real-time PCR	Increased *A. muciniphila* and decreased TNF-α mRNA expression.
Medina-Vera et al., 2018 [147]	81 patients with T2D divided into placebo and functional food-based diet (high fiber, polyphenol rich and vegetable protein) groups	Determination of fecal microbiota	Increased *F. prausnitzii* and *A. muciniphila* and decreased *P. copri*. Improvement in glucose, insulin, HOMA-IR, and LPS levels.
Elbere et al., 2018 [150]	18 healthy subjects were treated with metformin for 7 days	16S rRNA gene (V3 region)	Diversity of gut microbiota decreased (reduction of *Peptostreptococcaceae* and *Clostridiacea_1*) after metformin treatment.
Shimozato et al., 2017 [161]	66 T2D patients with and without chronic bowel movement disorder treated with placebo or transglucosidase	Analysis of fecal microbiota (amplification of 16S rRNA gene with T-RFLP)	Transglucosidase treatment modified the fecal microbiota (*Prevotella*, *Bacteroides*, *Bifidobacterium*, and the *Clostridium* sub-cluster XIVa) and the fecal SCFA and significantly improved bowel movements.
Canfora et al., 2017 [152]	Supplementation with galacto-oligosaccharides in 44 prediabetic patients	Fecal microbiota composition	Galacto-oligosaccharide supplementation increased *Bifidobacterium.*
Sato et al., 2017 [146]	Supplementation with *L. casei* in 68 T2D patients	Analysis of fecal microbiota	Probiotic administration increased *C. coccoides*, *C. leptum*, and *Lactobacillus.*
Mobini et al., 2016 [162]	46 patients with T2D on insulin therapy and *L. reuteri* DSM 17938 supplementation	Fecal microbiota composition	No changes in microbiota were observed.

**Abbreviations:** PCR: polymerase chain reaction; RCT: randomized clinical trial; mRNA: messenger RNA; rRNA: ribosomal ribonucleic acid; SCFA: short chain fatty acid; T2D: type 2 diabetes; TMAO: trimethylamine N-oxide; TNF-α: tumor necrosis factor alpha; TRFLP: terminal restriction fragment length polymorphism; V3 or V4: hypervariable 16S region.
